# Chemistry, Inorganic and Organic; with Experiments

**Published:** 1873-11

**Authors:** 


*Chemistry, Inorganic and Organic; with Experiments.* By Chas. Loudon Bloxam, Prof of Chemistry in Kings College, London, etc. From the Second Revised English Edition. With Two Hundred and ninety-five Illustrations. Philadelphia: Henry C. Lea, 1873. Buffalo: T. Butler & Son.

It is now five years since the first edition of this work was published, during that time the atomic system has been so generally adopted that the author has felt compelled to introduce it into the present edition. This of course has necessitated a large amount of labor and given the author at the same time an opportunity in the general revision to make such additions to, and corrections of the former text as time and experience have shown to be necessary. The author has endeavored as far as possible to retain the old nomenclature as there is not a general agreement upon the subject; the experimental character of the work has also been retained which will make it a favorite with those students who wish to observe for themselves. A copius index is a feature of the work, which will be received with general satisiaction. The illustrations are well made, and the general teachings of the work will be found to accord with the a.cepted views of the day.

